# Specimen mammography for intraoperative margin assessment in breast conserving surgery: a meta-analysis

**DOI:** 10.1038/s41598-022-23234-5

**Published:** 2022-11-02

**Authors:** Chen Lin, Kai-yue Wang, Hai-lang Chen, Yu-hua Xu, Tao Pan, Yi-ding Chen

**Affiliations:** 1 Department of Breast Surgery, The Second Affiliated Hospital of Zhejiang University School of Medicine, No. 88 Jiefang Road, Shangcheng District, Hangzhou, 310009 Zhejiang China; 2 Department of Oncology, Lanxi People’s Hospital, Jinhua, Zhejiang China

**Keywords:** Surgery, Radiography, Surgical oncology

## Abstract

In breast conserving surgery (BCS), specimen mammography is one of the most widely used intraoperative methods of assessing margin status. We performed a meta-analysis to evaluate the diagnostic accuracy of specimen mammography. Literature databases including PubMed, Cochrane Library, Web of Science, and EMBASE were searched prior to Jun 2022. A total of 1967 patients were included from 20 studies. A pooled analysis, heterogeneity testing, threshold effect testing, publication bias analysis, and subgroup analyses were performed from extracted data. The pooled weighted values were a sensitivity of 0.55 (95% confidence interval [CI], 0.47–0.63), a specificity of 0.85 (95% CI, 0.78–0.90), a diagnostic odds ratio of 7 (95% CI, 4–12), and a pooled positive likelihood ratio of 3.7 (95% CI 2.6–5.5). The area under the receiver operator characteristic curve was 0.75 (95% CI 0.71–0.78). In the subgroup analysis, the pooled specificity in the positive margin defined as tumor at margin subgroup was lower than the other positive margin definition subgroup (0.82 [95% CI: 0.71, 0.92] vs. 0.87 [95% CI: 0.80, 0.94], *p* = 0.01). Our findings indicated that specimen mammography was an accurate intraoperative imaging technique for margin assessment in BCS.

## Introduction

Breast cancer is one of the most common cancers worldwide. An important surgical modality for women with early-stage breast cancer is breast-conserving surgery (BCS). BCS entails removal of the cancer and sparing of the rest of the breast, balancing oncologic resection, and providing an ideal cosmesis. In recent years, more patients are diagnosed with smaller invasive tumors and are therefore likely to receive BCS. The use of neoadjuvant chemotherapy results in tumor downstaging, which leads to more patients undergoing BCS. Achieving a negative margin is critical in BCS, as a positive margin is one of the most significant risk factors for local recurrence. A positive margin occurs in 20–40% of all patients and 15–30% patients undergo reoperation to improve local control^1–4^. Reoperation is associated with a higher surgical risk, poorer cosmetic outcome, and increased psychological and economic burden^5^.

Histopathology is the gold standard for margin assessment. However, the analysis is performed post-operatively. Intraoperative margin assessment methods have been developed to identify positive margins and to enable immediate re-excision, thereby decreasing the reoperation rate. These methods mainly consist of two types, pathological methods, and imaging methods. Intraoperative pathological assessment methods, such as frozen section and imprint cytology, are time-consuming^6^. Intraoperative imaging methods have emerged aiming at real-time intraoperative management^7^.

Specimen mammography is usually used to confirm the excision of the targeted lesion. It is also an intraoperative resection margin assessment method performed during BCS. Two kinds of systems mainly exist in performing specimen mammography, that is, conventional specimen radiography (CSR) and intraoperative digital specimen mammography (IDSM). CSR is a widely used system in which the specimen is transported from the operating room to the radiology department for specimen radiography. CSR also requires compression of the surgical specimen between mammography plates which may impair its diagnostic accuracy^8^. Another system, IDSM, is developed by using a small automatic device placed near the operation room to take images, which helps to save operation time. Specimen compression is not needed in IDSM. Multiple views can be obtained and specimens can be placed with the proper orientations, as IDSM is often performed by the operating surgeon. IDSM may have some potential advantages, such as less anesthesia, less cost, predictable operating room schedule, reduced re-excision rate, and less tissue excised^9–11^.

Specimen mammography is widely used to assess margin status intraoperatively. However, its diagnostic accuracy remains controversial. Some studies have shown that specimen mammography was reliable in identifying clear margins and reduced the rate of reintervention^12^, while others have indicated it to be not accurate enough^13,14^. We performed this meta-analysis aiming at evaluating pooled diagnostic accuracy for specimen mammography in assessing margin status during BCS.

## Methods

Our study was conducted following guidelines of the “Preferred Reporting Items for Systematic Reviews and Meta-Analyses” (PRISMA)^15^. We searched relevant studies in Pubmed, Cochrane Library, Web of Science, and EMBASE prior to Jun 2022. The search strategy comprised both keywords and MeSH terms for “breast cancer” (MeSH term of Breast Neoplasms, breast cancer*, breast tumour*, breast tumor*, breast neoplasm*, Breast carcinoma*, mammary cancer*, mammary tumour*, mammary tumor*, Mammary Neoplasm*, mammary carcinoma*), “specimen mammography” ((mammograph* OR radiograph* OR X-ray) AND specimen*) and “margin*.” All types of articles were included. Extensive crosschecking of the references in retrieved articles was performed. Two investigators (Chen Lin and Kaiyue Wang) screened the titles and abstracts independently. All relevant citations were selected for further analysis. When a disagreement between two investigators regarding article inclusion surfaces, they discussed these articles with a senior author (Tao Pan). All three authors are surgeons.

Studies were included when they met the following criteria: (1) patients were diagnosed with breast cancer (invasive or in situ) and received BCS; (2) specimen mammography was performed intraoperatively to assess the margin status; (3) studies provided available data of true positive (TP), true negative (TN), false positive (FP), and false negative (FN). Studies not written in English were excluded. Opinions, case studies, reviews, conference abstracts, and meta-analyses were also excluded. Full-texts of the eligible studies were reviewed. The following information was extracted: (1) the first author; (2) year of publication; (3) number of patients; (4) number of lesions; (5)study design; (6) mean age of patients; (7) histopathological definition of positive margin distances; (8) positive margin rates; (9) mammography system used in the article; (10) diagnostic accuracy raw data- TP, TN, FP, and FN; and (11) percentages of sensitivity, specificity, positive predictive value, and negative predictive value.

Quality Assessment of Diagnosis Accuracy Study (QUADAS-2) form was used to assess methodological quality of the eligible studies^16^. QUADAS-2 checklist was independently completed by the two investigators (Hailang Chen and Yuhua Xu).

The relationship between sensitivity and specificity was assessed using a hierarchical summary receiver operating characteristic (SROC) model. The area under the curve (AUC) of the SROC was calculated to measure the diagnostic performance of specimen mammography. An AUC between 0.9 and 1.0 was considered a very good degree of diagnostic accuracy, between 0.7 and 0.9 represents a moderate degree and an AUC close to 0.5 AUC implies a poor degree^17^. The Youden index (*Q), which is used in conjunction with SROC analysis and recognized as a preferred statistic to reflect the diagnostic value, was also assessed. A *Q index of 1 indicates a perfect test result. Heterogeneity was assessed using I^2^. 25–49% was considered low level, 50–74% was moderate, and > 75% was high. A random effects model was used when I^2^ > 50%, and a fixed effects model was chosen when I^2^ < 50%^18^. The threshold effect was one of the important sources of heterogeneity in diagnostic accuracy tests. The Spearman correlation coefficients can determine the existence of the threshold effect if the p-value ≤ 0.05. Publication bias was analyzed using Deeks’ funnel plot. The absence of a non-zero slope coefficient (*P* < 0.10) indicates that a publication bias exists among the included studies^19^. A subgroup analysis was performed. Statistical software package Revman 5.3 and Stata version 16.0 (StataCorp, College Station, Texas) were used.

## Results

### Search results and study selection

The systematic search and manual crosschecking of references yielded 1255 articles from PubMed, Web of Science, Embase, and Cochrane initially. After duplicates were removed, 750 unique records remained. After excluding the obviously unrelated articles by reading the titles and abstracts, 93 remained as potential articles. An additional of 73 articles were excluded after a careful full-text review. The reasons for exclusion were as follows: (1) TP, TN, FP, and FN data were missing; (2) the paper did not focus on specimen mammography for margin assessment; (3) the paper was published in abstract format only; (4) the paper was a literature review; and /or (5) the paper was not written in English. Eventually, 20 studies were enrolled in the analysis^12,20–38^.

### Study description

**Figure 1:**
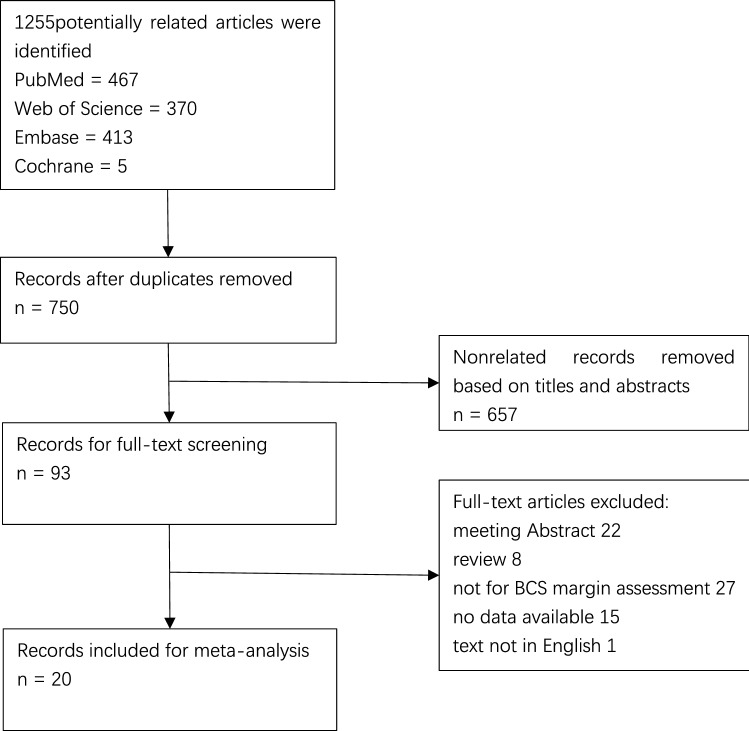
Flowchart illustrating the selection of the studies.

Our meta-analysis included 20 studies which consisted of a total of 1967 patients and 1988 lesions (Fig. 1) . The sample size ranged from 34 to 267 patients and 34 to 280 lesions. The mean or median age was available in 12 studies with a range between 50 and 60 years old. Sixteen studies mentioned the system used to take specimen mammograph. Eleven of the studies used the CSR system, three used the IDSM system and two used both systems for margin status assessment in BCS. Four studies did not provide information about the applied system. In seven studies, radiologists were blinded to the pathological results. The basic information and principal characteristics of the included studies including the number of patients and lesions, method of studies, mean age of patients, negative margin distance, positive margin rates, and mammography system are detailed in Table 1. Documented or calculable percentages of sensitivity, specificity, positive predictive value, and negative predictive value for each study are listed in Table 2. According to QUADAS-2 items, the quality assessment of 20 studies was moderate. The results of the distribution are shown in Supplementary Fig. S1.

**Table 1:** Principal characteristics of the included studies.

Author	Year	Number of patients	Number of lesions	Method of studies	Mean age	Negative margin distance	Positive margin rate	Mammography system
Yun, B. et al.^20^	2021	104	105	Retrospective	50	1 mm	19.05%	CSR
Lin, C. et al.^21^	2020	202	205	Retrospective	NS	0 mm	18,05%	IDSM
Mariscotti et al.^22^	2020	267	280	Retrospective	NS	0 mm	20.71%	Both
Park et al.^23^	2019	98	99	Retrospective	60	0 mm	14.14%	IDSM
Naz, S. et al.^25^	2018	52	52	Retrospective	51	0 mm	42.31%	NS
Pop et al.^24^	2018	83	83	Prospective	57	0 mm	10.84%	NS
Miller et al.^26^	2016	44	44	Prospective	57	0 mm	18.18%	Both
Hisada et al.^27^	2016	141	141	Retrospective	55	5 mm	20.57%	CSR
Chagpar et al.^28^	2015	90	90	Prospective	60	NS	37.78%	CSR
Layfield, DM et al.^29^	2012	104	104	Retrospective	60	2 mm for IDC, 5 mm for DCIS	29.81%	IDSM
Bathla et al.^30^	2011	99	102	Retrospective	59	0 mm	40.20%	CSR
Weber et al.^31^	2008	35	35	Retrospective	58	1 mm	57.14%	CSR
Ciccarelli et al.^12^	2007	102	102	Retrospective	NS	NS	37.25%	CSR
Coombs et al.^33^	2006	52	52	Retrospective	58	5 mm	44.23%	NS
Goldfeder, S. et al.^32^	2006	112	112	Prospective	NS	NS	43.75%	CSR
Łuczyńska, E. et al.^34^	2005	70	70	NS	NS	NS	45.71%	NS
McCormick, et al.^35^	2004	93	93	Retrospective	NS	NS	11.83%	CSR
Gombos, E. V.^36^	2004	34	34	NS	NS	NS	35.29%	CSR
Saarela et al.^37^	2001	66	66	Prospective	55	0 mm	40.91%	CSR
Graham et al.^38^	1994	119	119	NS	NS	1 mm	84.03%	CSR

*NS* not shown; *IDC* invasive ductal carcinoma; *DCIS* ductal carcinoma in situ; *IDSM* intraoperative digital specimen mammography; *CSR* conventional specimen radiography.

**Table 2:** Included studies containing raw diagnostic accuracy data for meta-analysis.

Author	Year	Number of lesions	TP	FP	FN	TN	Sensitivity (%)	Specificity (%)	PPV (%)	NPV (%)
Yun, B. et al	2021	105	11	20	9	65	55.00%	76.47%	35.48%	87.84%
Lin, C. et al	2020	205	24	10	13	158	64.86%	94.05%	70.59%	92.40%
Mariscotti et al	2020	280	27	65	31	157	46.55%	70.72%	29.35%	83.51%
Park et al	2019	99	14	61	0	24	100.00%	28.24%	18.67%	100.00%
Naz et al	2018	52	16	5	6	25	72.73%	83.33%	76.19%	80.65%
Pop et al	2018	83	4	11	5	63	44.44%	85.14%	26.67%	92.65%
Miller et al	2016	3	3	6	32	3	33.33%	91.43%	50.00%	84.21%
Hisada et al	2016	141	6	6	23	106	20.69%	94.64%	50.00%	82.17%
Chagpar et al	2015	90	14	12	20	44	41.18%	78.57%	53.85%	68.75%
Layfield DM et al	2012	104	18	14	13	59	58.06%	80.82%	56.25%	81.94%
Bathla et al	2011	102	24	5	17	56	58.54%	91.80%	82.76%	76.71%
Weber et al	2008	35	12	6	8	9	60.00%	60.00%	66.67%	52.94%
Ciccarelli et al	2007	102	25	9	13	55	65.79%	85.94%	73.53%	80.88%
Coombs et al	2006	52	12	4	11	25	52.17%	86.21%	75.00%	69.44%
Goldfeder et al	2006	112	24	17	25	46	48.98%	73.02%	58.54%	64.79%
Łuczyńska et al	2005	70	13	0	19	38	40.63%	100.00%	100.00%	66.67%
McCormick et al	2004	93	6	10	5	72	54.55%	87.80%	37.50%	93.51%
Gombos et al	2004	34	11	0	1	22	91.67%	100.00%	100.00%	95.65%
Saarela et al	2001	66	9	8	18	31	33.33%	79.49%	52.94%	63.27%
Graham et al	1994	119	62	1	38	18	62.00%	94.74%	98.41%	32.14%

*TP* true positive; *FP* false positive; *FN* false negative; *TN* true negative; *PPV* positive predictive value; *NPV* negative predictive value.

### Meta-analysis

The statistical results confirmed a moderate and high heterogeneity of specimen radiography for sensitivity (I^2^ = 65.48%) and specificity (I^2^ = 93.05%), respectively. A random effects coefficient binary regression model was used. The pooled estimate of 20 studies demonstrated a sensitivity of 0.55 (95% CI, 0.47–0.63), a specificity of 0.85 (95% CI, 0.78–0.90) (see Supplementary Fig. S2), and a diagnostic odds ratio of 7 (95% CI, 4–12). The pooled positive likelihood ratio was 3.7 (95% CI 2.6–5.5) and the pooled negative likelihood ratio was 0.52 (95% CI 0.44–0.62). The AUC value, which represented the overall diagnostic accuracy of specimen radiography, was 0.75 (95% CI 0.71–0.78). The *Q index was 0.6929 ± 0.0380. The SROC graph with the 95% confidence region and 95% prediction region is shown in Supplementary Fig. S3.

The Spearman correlation coefficients’ *p* values (0.779, *p* > 0.05) disclosed the absence of a threshold effect. Deeks’ funnel plot asymmetry test was performed and no significant publication bias (*p* = 0.26) existed in the enrolled studies (see Supplementary Fig. S4).

The results of the subgroup analysis were presented in Table 3 and Supplementary Fig. S5. BCS had been performed for decades. In the early period, there was no consensus on what constitutes an optimal negative margin width^39^. The various definitions of negative margin included "no tumor cells on the ink", no tumor cells seen at < 1 mm, < 2 mm, and larger distances (> 5 mm)^40^. The negative margin distances varied among the included studies. The studies were divided into two subgroups. All studies with a negative margin distance of 0 mm were included in the no tumor at margin subgroup. The remaining studies were included in the other negative margin definition subgroup. The subgroups had comparable pooled sensitivity (0,58 [95% CI: 0.45, 0.71] vs. 0.53 [95% CI: 0.43, 0.64], *p* = 0.95), but the pooled specificity was significantly lower in the no tumor at margin subgroup (0.82 [95% CI: 0.71, 0.92] vs. 0.87 [95% CI: 0.80, 0.94], *p* = 0.01).

**Table 3:** Univariable meta-regression and subgroup analysis.

Subgroup	Category	Number of studies	Sensitivity with 95% CI	*p* value of Sensitivity	Specificity with 95% CI	*p* value of Specificity
Positive margin rate > 40%	Yes	8	0.54 (0.42–0.66)	0.56	0.87 (0.78 – 0.96)	0.13
	No	12	0.56 (0.45–0.67)		0.84 (0.77 – 0.92)	
Only conventional specimen radiography	Yes	11	0.53 [0.42–0.64]	0.38	0.86 [0.78 – 0.94]	0.25
	No	9	0.58 [0.46–0.71]		0.84 [0.75 – 0.94]	
Number of lesions > 100	Yes	9	0.54 [0.42–0.65]	0.51	0.87 [0.79 – 0.94]	0.17
	No	11	0.57 [0.45–0.69]		0.84 [0.75 – 0.93]	
Prospective study	Yes	5	0.40 [0.25–0.54]	0.13	0.83 [0.70 – 0.96]	0.11
	No	15	0.60 [0.51–0.68]		0.86 [0.79 – 0.93]	
Blind	Yes	7	0.55 [0.42–0.69]	0.71	0.89 [0.81 – 0.97]	0.28
	No	13	0.55 [0.45–0.65]		0.83 [0.75 – 0.91]	
Negative margin defined as no tumor at margin	Yes	8	0.58 [0.45–0.71]	0.95	0.82 [0.71 – 0.92]	0.01
	No	12	0.53 [0.43–0.64]		0.87 [0.80 – 0.94]	

## Discussion

Histopathological results of the margin status occupy a particularly important place in BCS. The application of an accurate intraoperative diagnostic method can reduce the reoperation rate. In recent years, numerous studies have compared the ability of techniques in detecting positive margins to the final histopathological results. Assessing the diagnostic accuracy of intraoperative methods is necessary.

Intraoperative pathological and imaging methods are the two main methods for intraoperative margin assessment in BCS^41^. Intraoperative pathological methods mainly include frozen section, touch smear, and imprint cytology, which all have high sensitivity, specificity, and accuracy^42^. Despite the high level of accuracy, intraoperative pathological techniques are infrequently employed and depend heavily on the pathologists’ experience. Pathological methods are also time-consuming. They often add an average of 20–30 additional min to the operation time^6^. Intraoperative imaging methods can quickly assess margin status and reduce operation time. Specimen mammography is one of the most widely used intraoperative imaging methods. Various studies are available in the literature to evaluate the value of specimen mammography in margin status assessment. The findings of our meta-analysis indicated that the diagnostic accuracy of specimen mammography was promising, with an AUC of 0.75, despite some limitations.

There was moderate and high heterogeneity for sensitivity and specificity, respectively, in our meta-analysis. The heterogeneity among studies may be attributed to factors, such as study design, type of specimen mammography system, and different definitions of negative margin. Two types of systems mainly exist which are used to take specimen mammography, namely, CSR and IDSM. A two-dimensional CSR is a technique used routinely in many cancer institutions. The device used for performing CSR is usually is the same one used for patients’ mammography. The device is often located in a radiology unit. Surgical specimen needs to be transported from the operating room to the radiology department, which makes it labor-intensive and time-consuming. The disadvantages of CSR also include low specificity, as additional excision of the tissue is often recommended unnecessarily^43^. Specimens examined with CSR are often compressed with a plate, to simulate the conditions of the mammographic examinations^22^. Kyle Ota believed that the high false positive rate may be caused by “pancake phenomenon^43^”, where CSR compresses the surgical specimen. This manipulation refers to a reduction in the mean volume and height of the breast specimens which may increase FP margins^8^. The other widely used system is IDSM. It is a portable, dedicated, self-contained digital imaging system, often placed near the operation room. Without transporting the specimen to the radiology department, IDSM shortens the specimen transport time. IDSM results can be read by the surgeon near the operation room. Real-time review shortens the time required for radiologist review. When the radiologist is not immediately available, the surgeon can review images and make decisions. However, it acquires additional skills in learning image interpretation. The IDSM system significantly reduced the operative time for BCS compared to CSR^26^, which in turn decreased anesthesia time and operation room cost. Aside from the reduced time, some studies have found a reduction in the reoperation rates after the introduction of IDSM^12,22^. However, in our study, no significant difference was observed between subgroups of CSR and IDSM. Further assessment requires large-scale and well-designed clinical trials.

Different definitions of negative margin may also lead to heterogeneity among studies. Before 2014, controversy exists regarding the optimal margin width in BCS for breast cancer^40^. The American Society of Clinical Oncology guidelines suggested that tumor margins not touching ink at the specimen edge were acceptable for both invasive cancer and ductal carcinoma in situ^44,45^. However, a recent study indicated that close margins less than 2 mm were associated with increased local and distant recurrence as well as lower overall survival, compared with wide margins^46^. Researches are still undergoing to explore an optimum margin width, in order to pursuing lower recurrence rate and better cosmetic outcome. Different margin widths exist among countries and institutions using different guidelines. Among the included studies, a negative margin width differs from no tumor at the margin to no tumor within 5 mm from the margin^40^. A wider negative margin distance defines those cases with close margins as pathologically positive. “Pancake phenomenon” causes the reduction in mean volume and height of the breast specimens, which also tends to define close margin cases as radiological positive^43^, leading to better diagnostic accuracy in the wider negative margin subgroup. Our study showed a significant difference in specificity between the two subgroups with higher specificity in the group with wider negative margins.

Besides the factors mentioned above, there might be other factors affecting the diagnostic accuracy of specimen mammography. Goldfeder found that concordance between specimen radiography and histopathology was higher in one-view specimen mammography in comparison to two-view^32^. Mammographs of specimens were more effective in evaluating the surgical margins of mammographic lesions with microcalcifications than other manifestations^47^. In some subtypes of breast cancer such as medullary carcinoma, the positive margin signs on specimen mammograph might not represent actual tumor but only a nonneoplastic infiltrate of lymphocytes^48^.

Other important limitations of intraoperative specimen mammography are: (1) specimen mammography is a two-dimensional imaging method which is inherently flawed in depicting three-dimensional specimens comprehensively^41^; (2) because of the poor soft tissue contrast, the difficulty in determining the margin status using specimen mammography increases with the breast density^49^.

Emerging techniques have been developed to improve those limitations. Vacuum intraoperative specimen mammography has been applied to increase the precision of margin detection by decreasing compression, as specimen compression may affect the evaluation of tumor borders^50^. Three-dimensional imaging techniques, such as digital breast tomosynthesis and micro-computed tomography, have been introduced to solve the dilemma essentially^23,51,52^. Our meta-analysis data demonstrated a sensitivity and specificity of 0.55 (95% CI, 0.47–0.63) and 0.85 (95% CI, 0.78–0.90), respectively. With a relatively low sensitivity, specimen mammography is less accurate than pathological methods. However, despite those limitations, intraoperative specimen mammography is a widely used and cost-effective procedure for margin assessment. It also helps to reduce the operation time. Specimen mammography can aid in the intraoperative assessment of margin status. Overall, with an AUC value of 0.75, our findings indicated intraoperative specimen mammography to be accurate in assessing margin status during BCS.

## Supplementary Information

Supplementary Information.

## Data Availability

All data generated or analyzed in this study are included in this published article and its supplementary files.
